# Appraising and comparing the role of autogenous periosteal graft as a barrier membrane in the treatment of intrabony defects in chronic periodontitis cases: A systematic review and meta-analysis

**DOI:** 10.7150/ijms.86720

**Published:** 2024-01-01

**Authors:** Jui Bhandare, Swapna A. Mahale, Saquib S. Abullais, Ankita Katkurwar, Saad M. AlQahtani, Youssef A Algarni, Shaik Mohammed Asif

**Affiliations:** 1 Department of Periodontology, MGV's KBH Dental College and Hospital, Nashik, 422003, India.; 2 Department of Periodontics and Community Dental Sciences, King Khalid University, Abha, 61421, KSA.; 3 Department of Restorative Dental Sciences, College of Dentistry, King Khalid University, Abha 61321, Saudi Arabia.; 4 Department of Diagnostic Science and Oral Biology, College of Dentistry, King Khalid University, Abha 61321, Saudi Arabia.

**Keywords:** chronic periodontitis, intrabony defects, autogenous periosteal graft, meta-analysis, systematic review

## Abstract

Periodontal regeneration refers to procedures aimed at restitution of lost supporting tissue around the periodontally compromised tooth. Regenerative procedures very often include the use of barrier materials to encourage the growth of key surrounding tissues. The current study aimed to evaluate the effectiveness of autogenous periosteal graft as a barrier membrane for the treatment of intrabony defects in chronic periodontitis patients. A total of four data bases MEDLINE (by PubMed), Cochrane database, EBSCO, and Google Scholar were explored to identify the studies in English up to December 2022. An additional hand search of relevant journals was also done. A team of three independent reviewers screened the retrieved articles using the inclusion criteria. Randomized control trials (RCTs) evaluating the effectiveness of autogenous periosteal grafts in the treatment of intrabony defects in chronic periodontitis cases were included in the study. A total of six relevant articles were recognized for data procurement. A total of 117 patients with 68 sites with an age range between 18 years and 55 years were selected. Outcome variables examined were pocket depth (PD), clinical attachment level (CAL), radiographic bone defect fill (BDF), gingival recession (GR), plaque index (PI), gingival index (GI) and bleeding on probing (BOP). Data were analyzed using Revman 5.3 software. The mean differences and 95% confidence interval were used to illustrate the estimate of effect size. There is an equal effect in both groups for the PI, GI, and BOP reduction. For PD reduction, the result was in the favor of periosteal graft with open flap debridement (OFD) group. For CAL gain, radiographic BDF and GR, results also favored the periosteal graft, but no statistically significant difference was found amongst the groups. Within the limitation of the study, it seems that the autogenous periosteal graft can be used successfully along with OFD to treat intrabony defects in chronic periodontitis patients.

## Introduction

Periodontal regeneration refers to the restoration of bone, cementum and periodontal ligament to their original levels once damaged by periodontal disease. It has been shown that periodontal regeneration can be achieved by a variety of non-surgical and surgical procedures 1. Surgical modalities of periodontal regeneration include osseous grafts, barrier membrane materials and a combination of both 2. Osseous graft techniques, although effective in decreasing probing depths and improving attachment levels, do not promote true regeneration of the periodontal unit on a predictable basis 3. Often, a new junctional epithelium is present between the regenerated alveolar bone and the previously diseased root surface 4.

Attempts to regenerate interproximal defects with currently available barrier membranes have been somewhat less than optimal 5. Barrier membranes serve the purpose of preventing epithelial downgrowth, thereby allowing progenitor cells of bone and periodontal ligament to regenerate desirable tissues from the base of the defect 6. If barrier membranes become exposed, it will lead to the accumulation of bacterial plaque within the interproximal defect resulting in impaired healing 7.

Several non-resorbable and bioabsorbable materials have been used as barrier membranes in the GTR procedure 8. Among these, autogenous periosteal grafts, as both free and pedicle grafts, have been proposed for the treatment of periodontal defects 9. Indeed, several studies have reported successful clinical outcomes for regenerative treatment of intrabony and furcation defects by autogenous periosteal barrier membranes, compared to control sites treated by open-flap debridement (OFD) alone 10.

The effectiveness of the simultaneous use of a barrier membrane and a filling material, as a combined periodontal regenerative technique (CPRT), has been evaluated previously in different types of periodontal lesions (i.e., intrabony defects and furcation defects) and compared to GTR procedures with membranes alone; however, conflicting results have been obtained, particularly when dealing with the treatment of intrabony defects 11.

Factors related to the morphology of a bone defect, such as the total depth of the intraosseous component and the radiographic angle of the defect, can significantly affect the clinical outcome of the GTR procedure 12. The architecture of a defect is related to the effectiveness of the remaining bone structure to sustain the barrier membrane. Thus avoiding the membrane collapse towards the bone defect by the pressure of the soft tissues is a crucial issue in regeneration failure. To avoid barrier-membrane collapse, the placement of filling materials underneath the membrane has been suggested 13.

Both non-resorbable and bioabsorbable membranes become problematic if exposed to the oral flora 14. This is very crucial in the case of interproximal bony defects due to the inability to achieve complete soft tissue coverage. It will lead to exposure of the membrane and increase the chances of infection at the initial period of healing. Autogenous periosteal grafts are an attractive alternative to existing barrier membrane materials since they meet the requirements of an ideal material and are biologically accepted 16. Moreover, the periosteum has the potential to stimulate osteogenesis in the bony defect area 17.

This systematic review and meta-analysis was intended to examine the viability of autogenous periosteal graft along with open flap debridement and open flap debridement alone for the treatment of intrabony defects in chronic periodontitis patients.

## Material and methods

### Protocol and Registration

To avoid any unintentional reiteration of the review on this topic, registration of the review protocol was done at an international database of prospectively registered systematic reviews PROSPERO (CRD42021258038). We composed the review as per Preferred Reporting Items for Systematic Review and Meta-analysis (PRISMA) guidelines 18 and also followed the PRISMA statement and Cochrane Handbook for Systematic Reviews of Interventions 19. The review question was: “What is the viability of autogenous periosteal membrane along with open flap debridement and open flap debridement alone in the treatment of intrabony defects in chronic periodontitis patients?”.

### Focused PICOS Question

The following PICOS model was employed for this review:

P-Chronic periodontitis patients with intrabony defects.

I-Interventions being evaluated was the surgical technique of open flap debridement with autogenous periosteal membrane.

C-Comparison was done with the surgical technique of only conventional open flap debridement.

O-Different types of the outcome being measured were

*Primary outcome:*

1. Pocket depth (PD)

2. Clinical attachment level (CAL)

3. Radiographic bone defect fill (BDF)

*Secondary outcomes:*

1. Gingival recession (GR)

2. Plaque index (PI)

3. Gingival index (GI)

4. Bleeding on probing (BOP)

In the included studies, all these clinical parameters were evaluated for a period of a minimum of 6 months and a maximum of 9 months postoperatively.

S-Studies searched were randomized control clinical trials (RCTs), published only in the English language and restricted to intrabony defect.

### Search Strategy

We executed a comprehensive literature search till December 2022. Extensive search strategies were instituted to analyze the studies for the present systematic review. Four electronic databases, namely MEDLINE (via PubMed), Google Scholar, Cochrane database and EBSCO databases were scrutinized for published articles. A broad search strategy was developed for MEDLINE: MeSH terms, keywords and other free terms will be wielded for exploring the base of evidence; Boolean operators (AND and OR) were utilized to combine searches. (periosteal pedicle graft OR periosteum as barrier membrane) AND (open flap debridement OR root debridement) (intrabony defect OR interproximal bony defect) (periodontitis OR periodontal diseases OR periodontal surgery OR chronic periodontitis patients) AND (randomized controlled trial OR RCT).

Reference lists of any potential articles and OpenGray database were scrutinized to investigate for potentially relevant unpublished studies or papers not identified by electronic searching. Additionally, the electronic database of four dental journals was scrutinized - namely: Journal of Periodontology, Journal of Clinical Periodontology, Journal of Periodontal Research and Journal of Indian Society of Periodontology. There was no time restriction applied for the article search, although human Randomized Controlled Trials published in the English language were included in the systematic review.

### Inclusion criteria and Exclusion criteria

Studies were screened based on titles and abstracts; if a decision could not be made based on this information, full papers were reviewed. Randomized controlled trials (RCT) with a duration ≥ 6 months were regarded as eligible for inclusion. Studies were included if they have revealed outcomes from chronic periodontitis patients possessing intrabony defects, who have been treated with either autogenous periosteal membrane after open flap debridement or open flap debridement alone. Pieces of literature were included if they met the following criteria:

Patients were included if:

1. Systemically healthy patients aged more than 18 years

2. Presence of probing pocket depth ≥ 5 mm following Phase I therapy

3. Patients who can maintain good oral hygiene

4. Both males and females were included.

Studies were excluded if:

1. Patients with a habit of smoking.

2. Pregnant or lactating patients.

3. Patient with poor oral hygiene.

4. Systemic problems affecting periodontal tissues.

5. Studies were excluded if they had insufficient data for pooling.

### Screening and Data Extraction

Three reviewers (J.B., S.M., A.K.) independently screened the title and abstract of the initially identified studies. Any duplication or articles that did not meet inclusion criteria were exempted. Full-text copy for all eligible articles was obtained and two reviewers (J.B., S.M.) assessed them separately to determine whether they qualify the inclusion norms. Any disagreement was resolved by discussion. Articles were excluded if they were not as per the inclusion norms. The reasons for exclusion were recited for justification. The data of the included studies were extracted in a Microsoft Excel sheet.

### Outcome Measurements

Primary Outcome:

- Change in pocket depth (PD) was reported as a gain in clinical attachment level at the 6-month or 9-month follow-up evaluation (PD was measured from the margin of free gingiva to periodontal pocket).
- Change in clinical attachment level (CAL) was reported as a reduction in recession at the 6-month or 9-month follow-up evaluation (CAL was referred to as the distance from the CEJ to the most apical part of the sulcus).
- Change in bone defect fill (BDF) was reported as an increase in the bone opacity on the radiograph

Secondary Outcome:

- Change in PI was reported as a reduction in plaque at the 6-month or 9-month follow-up evaluation
- Change in GI was reported as a reduction in gingival inflammation at the 6-month or 9-month follow-up evaluation
- Change in GR was reported as a change in the gingival marginal position coronal to its original position at the 6-month or 9-month follow-up evaluation (GR was measured from the marginal gingiva to CEJ).
- Change in BOP was reported as a reduction in bleeding upon probing at the 6-month or 9-month follow-up.

### Risk of Bias

Two investigators (J.B. and A.K.) investigated the quality of selected studies separately using the risk of bias assessment tool (The Cochrane Collaboration's tool) 19. If any debate over a review, then it was settled by vocal conversation. The studies were categorized as a high, low, or unclear risk of bias by using the risk of bias assessment tool. After the quality assessment, the included studies were graded into

(1) low risk: when all criteria were met or one criterion was unclear/ not met;

(2) moderate risk: when two criteria were unclear/not met;

(3) high risk: when more than two criteria were not met.

As per the Cochrane Handbook, Chi-square and Higgins index (I^2^) were used to decide the heterogeneity.

### Statistical Analysis

For the meta-analysis, Revman 5.3 (Review Manager Version 5.3; The Cochrane Collaboration, Copenhagen, Denmark) software was employed for the included studies. The continuous data (including PD, CAL, BDF and RW were estimated as mean difference (MD) and 95% confidence interval (CI), with p < 0.05 being statistically significant. The fixed-effect model was applied when the heterogeneity between the studies was low (p ≥ 0.10, I^2^ ≤ 50%) and when heterogeneity was high (p < 0.10, I^2^ > 50%), the random effect models were applied for meta-analysis. The heterogeneity across studies in PD, CAL, BDF and RW was correlated through subgroup analysis. The results of the meta-analysis were represented in the forest plot.

## Results

### Study selection

**Figure 1:**
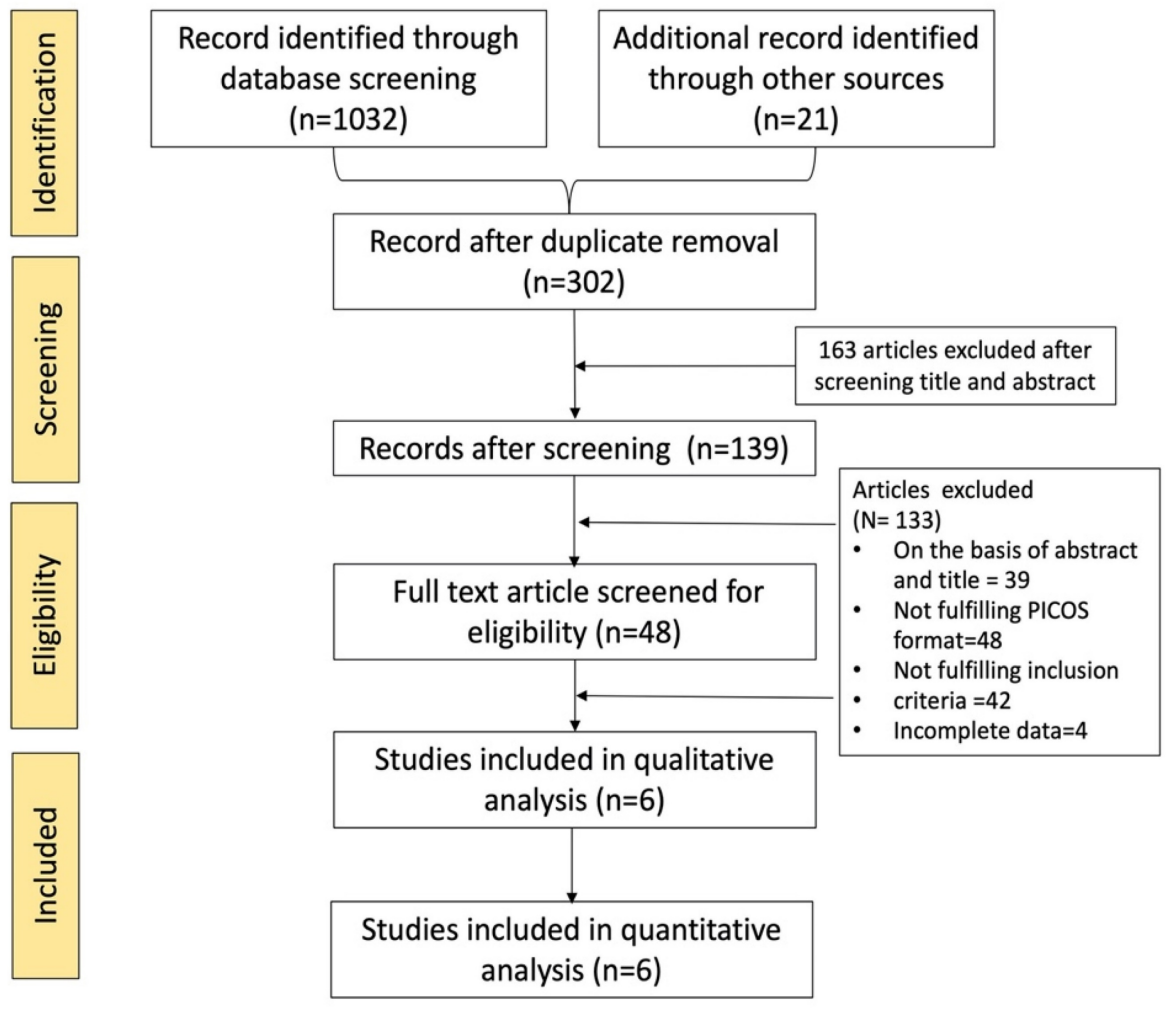
Process of study selection described in PRISMA flow diagram.

A total of 1032 records were identified through comprehensive database searching, whereas 21 records were collected from other sources. After careful examination and duplicate identification, 751 articles were eliminated which resulted in the remaining 302 articles. These 302 articles were further screened for title and abstract which resulted in the remaining 139 articles after excluding 163 articles. These 139 articles were scrutinized, 39 articles were excluded due to inefficient data in the title and abstract, 48 articles failed to fulfill the PICOS format, and four articles did not have complete data to be added for meta-analysis. Forty-eight full-text articles were assessed for eligibility, out of which 42 articles failed to meet the inclusion of the current study. Finally, 6 studies were selected for data extraction 10, 20-24. The selection process was outlined in the PRISMA flowchart (Figure 1) 25.

### Study characteristics

Six relevant articles were recognized for data procurement (Table 1) 16,20-24. Altogether 117 patients with 68 sites with an age range between 18 years and 55 years of participants were selected. Out of all patients, 88 males and 29 females were shortlisted for the systematic review. The characteristics of the included articles were illustrated and the extracted data were outlined in Table 2.

The primary factor for study selection was chronic periodontitis patients having intrabony defects treated with either autogenous periosteal graft as barrier membrane with open flap debridement or open flap debridement alone. The outcomes were objectively measured by its pocket depth, clinical attachment level and radiographic bone defect fill. Secondary study factors used to assess the outcomes were gingival recession, PI, GI, and BOP.

Phase I therapy was done in all the studies except one study 22, which included full mouth scaling and root planing for all teeth, which was performed in quadrants under local anesthesia. Two studies 16, 21 assessed open flap debridement with periosteum used as a barrier membrane in the intervention group, whereas one study 24 assessed the use of vascularized periosteum as an autogenous guided tissue regeneration membrane for defect coverage to evaluate the type of healing, wherein they did not mention if open flap debridement was performed. The study done by Kwan et al., assessed the periosteal connective tissue barrier in the intervention group 20.

Three of the studies 20, 22, 23 compared the intervention group to conventional open flap debridement, whereas Kumar et al., 16 and Singhal et al., 21 used periosteum as a barrier membrane along with alloplastic graft in comparison groups. The longest intervention period was 9 months by Gamal Y. et al., 23 and Kumar et al., 16 Furthermore, Ahmed et al., also intervened at 3, 6 and 9 months. Kwan et al., 20 Singhal et al., 21 and Ghallab et al., 24 completed the trial in 6 months. The radiographic investigation included radiovisiography (RVG) by Kumar et al., standardized intraoral periapical radiographs by Kwan et al.,^20^ Singhal et al.,^ 21^ and Gamal Y. et al. 23.

Medications prescribed by Kwan et al., 20 post-surgery were Oral antibiotics (penicillin VK 250 mg Q.I.D for 7 days) and 0.12% Chlorhexidine rinses (twice daily for 2 weeks) oral analgesics (ibuprofen 800 mg). Singhal et al., 21 recommended Doxycycline 100 mg twice on the first day, followed by 100 mg once daily for 5 days, and 0.2% chlorhexidine mouthwash for 1 minute twice daily. Gamal Y. et al., 23 gave Amoxicillin 500 mg TID for one week and chlorhexidine mouth-rinse for one minute (0.12%) 3 times daily for 1 month.

Kwan et al., 20 used Marquis periodontal probe whereas the other studies did not mention the type of periodontal probe used. The surgical procedure done by Kwan et al., 20 is described as buccal and lingual mucoperiosteal flaps done using intrasulcular incisions. Interproximal tissue was preserved in the flap design. The graft obtained was from the palatal area of a quadrant other than the one receiving treatment. Occlusal stents were used in all studies, however, Kwan et al., 20 made the occlusal stent cover the occlusal surface of the tooth being treated, as well as the occlusal surfaces of at least one adjacent tooth in the mesial and distal directions.

Suture removal was done at one week in all studies except in Ahmed Gamal Y. et al., study 23, where the suture removal was done at two weeks. Kumar et al., 16 Kwan et al., 20 and Singhal et al., 21 included the postoperative complications.

### Quality of studies

Only two out of six studies mentioned sequence generation: Ghallab et al., 24 used computer programs for sampling and Gamal Y. et al., 23 used the coin toss technique. The remaining four articles did not mention the method of random sequence generation.

Allocation and concealment were only seen in the study by Ghallab et al., 24 by placing this sequence in sequentially numbered, opaque, sealed envelopes which included the randomization code for each patient that was not broken until follow-up was concluded.

As these were surgical procedures, blinding the patients for the procedure was not possible, but in the study done by Ghallab et al., 24 the participants, outcome assessor and statistician were blinded to the type of intervention being allocated. All the studies had complete follow-up reports. None of the studies stated selective reporting and other biases.

On evaluation, one article 24 was claimed as low risk, one 23 was moderate risk and four articles as high risk 16,20, 22 (when three to four criteria were not met or unclear) for seven risk of bias criteria. Figure 2 summarizes the quality assessment of the included studies.

### Outcome

#### Probing pocket depth

All studies 20-24 except the study done by Kumar et al.,16 were included in the meta-analysis. A random-effects model was employed (I2 = 0%) since there was no reported heterogeneity. In all the studies, probing depth reductions were consistently greater in experimental sites; however, the differences were not statistically significant according to subgroup analysis (Figure 2A).

#### Clinical attachment level

A total of five studies 20-24 were included in the meta-analysis. Study done by Kumar et al.,16 was excluded as it did not provide specific data related to clinical attachment levels. The model employed was a random-effects model (I2 = 94%). All the studies reported clinical attachment level gain in experimental sites having significantly greater gain as compared to the control sites. The forest plot thus demonstrated high heterogeneity with the overall effect being highly significant (Figure 2B).

#### Bone defect fill

All the studies 16, 20-24 were included in the meta-analysis. A random-effects model was employed (I2 = 99%) which reported high heterogeneity. The study done by Kumar et al., 16 assessed linear measurements of the distance from the cementoenamel junction to the base of the bone defect (CEJ-BBD) through the radiograph. It showed that CEJ-BBD distance showed no statistically significant intergroup difference. In one study 20 reported that defect fill was seen to be more apparent in the sites treated with periosteal graft as a barrier membrane. There was no statistical significance observed within all the groups (Figure 2C).

#### Gingival recession

The meta-analysis was performed on all six 16, 20-24 studies increased in the gingival recession. A model employed (I^2^ = 39%) was a random-effects model. The results manifested the use of periosteal graft as a barrier membrane in the treatment of gingival recessions. As per the results of the subgroup analysis, only OFD could not improve the gingival recession. No significant difference was not found in the subgroup of periosteal graft and OFD groups (Figure 2D).

#### Plaque index

A total of five studies 20-24 were included in this meta-analysis including the plaque index. One study was excluded as they did not perform plaque index 16. The model employed was a random-effects model (I2 = 0%). The results revealed that there was no statistically significant difference in the plaque index as observed in all three of the studies (Figure 2E).

#### Gingival index

Out of all studies, only two studies those conducted by Gamal Y. et al., 23 and Singhal et al., 21 assessed the Gingival index. Other studies did not assess this parameter 16, 20, 22, 24. It showed that there was no statistically significant difference in the gingival index in comparison to both test and control groups. A random-effects model was employed (I2 = 0%) since there was no reported heterogeneity. All the included studies showed a significant decrease in the gingival inflammation hence leading to decreased gingival index; however, there was no significant difference in the intergroup comparison ((Figure 2F).

#### Bleeding on probing

Two studies 20, 23 out of six were excluded from meta-analysis as there was incomplete data. As the heterogeneity was high (I2=98.4%) a random-effect model was employed. According to the results of our meta-analysis, the sites treated with periosteal graft as a barrier membrane showed lesser bleeding on probing on follow-up of 6 months and then 9 months. Subgroup analysis exhibited a significant difference for the periosteal graft group as compared to the OFD group, but for the OFD group, no significant difference was observed (Figure 2G).

## Discussion

Periodontal regeneration refers to the restoration of bone, cementum and periodontal ligament to their original levels once damaged by periodontal disease 26. It has been shown that periodontal regeneration can be achieved by a variety of non-surgical and surgical procedures Regenerative periodontal therapy aims to predictably restore the tooth-supporting periodontal tissues (i.e. new periodontal ligament, new cementum by inserting periodontal ligament fibers and new bone) that have been lost due to periodontal disease 27, 28. Several modalities of periodontal regeneration include the incorporation of osseous grafts, guided tissue regeneration (GTR), or the combination of both 29. Bone grafting is the most common form of regenerative therapy although effective in decreasing probing depths and improving attachment levels, which do not promote regeneration of the periodontal unit on a predictable basis 30.

Barrier membranes prevent epithelial down growth and allow progenitor cells of the periodontal ligament to regenerate the tissues directly from the base of the defect 31. Autogenous periosteal grafts are an attractive alternative to existing barrier membrane materials since they meet the requirements of an ideal material and are biologically accepted. Moreover, the periosteum is highly vascular and known to contain fibroblasts and their progenitor cells i.e. osteoblasts and stem cells 32. The cells of the periosteum retain the ability to differentiate into fibroblasts, osteoblasts, chondrocytes, adipocytes, and skeletal myocytes. The tissue produced by these cells includes cementum with periodontal ligament fibers and bone 33. Therefore, we compared the open flap debridement with or without autogenous periosteal graft in the treatment of intrabony defects in chronic periodontitis patients.

Only randomized controlled trials were scrutinized for this systematic review to avoid any methodological inadequacy and to get better and improved evidence. The reduction in the intrabony defect depth shows the highest success rate with periosteal pedicle graft as a barrier membrane 34. Thus, the studies having chronic periodontitis patients with the existence of intrabony defects were included. Smokers were eliminated in all the studies as they negatively influence the gain in CAL, reduction in plaque and gingival recession, which could depreciate the results after surgical intervention 35.

There was an equal outcome effect in both the groups for the PI, GI and BOP reduction. But in the case of bone defect fill, the results were in the favor of periosteal barrier membrane with the OFD group, but the difference was not statistically significant. The variance in heterogeneity in bone defect fill was observed. This could be associated with different surgical techniques performed in the studies 16, 22-24 where the autogenous periosteum was attached at one site to the mucoperiosteal flap to maintain its vascular supply. Pedical mucoperiosteal flap is important for healing and maintaining the vital cell layer, that has the potential to stimulate bone formation as juxtaposed to exposed intrabony defect. This was in accordance with the study done by Mahajan et al., 35. Kwan et al., 20 in which they harvested the periosteum with connective tissue from the palatal site as a biological barrier membrane. Since, it has an optimum regenerative capacity and better healing, which was later confirmed by Elfana et al., 37 in their research.

In one study, 20 the mean bone defect fill and probing depth was statistically significant however, it was not significant as compared to the other studies 16, 21-25 This could be because the pedicle biologic barrier membrane provides a better result than free tissue transfer. This could be attributed to several things related to the outcome as there was no additional effect on the healing as well as the direct lateral positioning of the membrane 38. It has been reported that bone formation is affected by the degree of surgical damage to the periosteum and the form of the periosteum while harvesting it 39.

The results with autogenous periosteum as a GTR barrier membrane showed the formation of an osseous structure resulting in adequate bone defect fill. The osteogenic potential of periosteum discussed by Abu-Shahba et al., 40 may explain the differences in defect fill between the control and test groups showing statically significant results in all the studies. Hirata et al., 41 described the ability of vascularized periosteum to form new bone, which can be correlated to the statistically significant bone defect fill. All the studies 16, 20-24 included in this systematic review demonstrated a substantial increase in bone density clinically and radiographically. In five studies, the autogenous periosteal membrane maintained its vascular supply as it was attached on one side to the mucoperiosteal flap which helps in the healing and maintenance of the vital cambium layer which has the potential to stimulate bone formation.

In the pocket area, the periosteum may get infected and destroyed If the pocket depth is 5 mm or more in the defect area, 42 then the periosteal barrier membrane becomes difficult to position to the full extent. This explained a significant reduction in the pocket depth in the study done by Kwan et al., 20 where they used a free periosteal membrane. For BOP reduction and clinical attachment level gain, a statistically significant difference was found in the study where periosteal pedicle graft was used along with OFD as a barrier membrane which was similar to the result found in all the mentioned studies. In terms of GR gain exhibited effective results along with OFD but the difference was not statistically significant. Heterogeneity in GR seems to be because of different techniques employed for obtaining the periosteal pedicle graft. The mean decrease in PI score, GI score and change in gingival recession was not found to be significant, which was similar to the study demonstrated by Lekovic et al., 43.

### Limitations

The limitations of the systematic review could be that histologic evaluation is needed to confirm the efficacy of the periosteal membrane in promoting true periodontal regeneration. The sample size was relatively small for all the studies. The duration of the study was about 6 to 9 months. Studies with long-term follow-up are required to formulate strong evidence. Future studies should maintain 2-5 years of follow-up to get stable results of this technique. Only six articles were included in this systematic review and meta-analysis, as many articles were excluded as the criteria were not matching and due to insufficient data available. If more articles are included in the analysis, they will generate more significant results. It is important to consider that the results of this systematic review may be affected significantly by other factors such as tension on the membrane, the thickness of the connective tissue, the viability of the periosteum, width of the defect, maintenance of oral hygiene and experience of the operator.

## Conclusion

Thus, within the limitation of the study, it seems that the autogenous periosteal pedicle graft can be used efficaciously along with OFD as equated to OFD alone for the treatment of intrabony defects in chronic periodontitis. However, only a speculative inference can be drawn from this study since there is an inadequate number of studies with restricted data, a smaller sample size, a follow-up period of 6 months duration, and a relatively high risk of bias. Hence, higher quality RCTs with longer follow-up and substantial sample size are needed to draw a definitive conclusion.

## Figures and Tables

**Figure 2:**
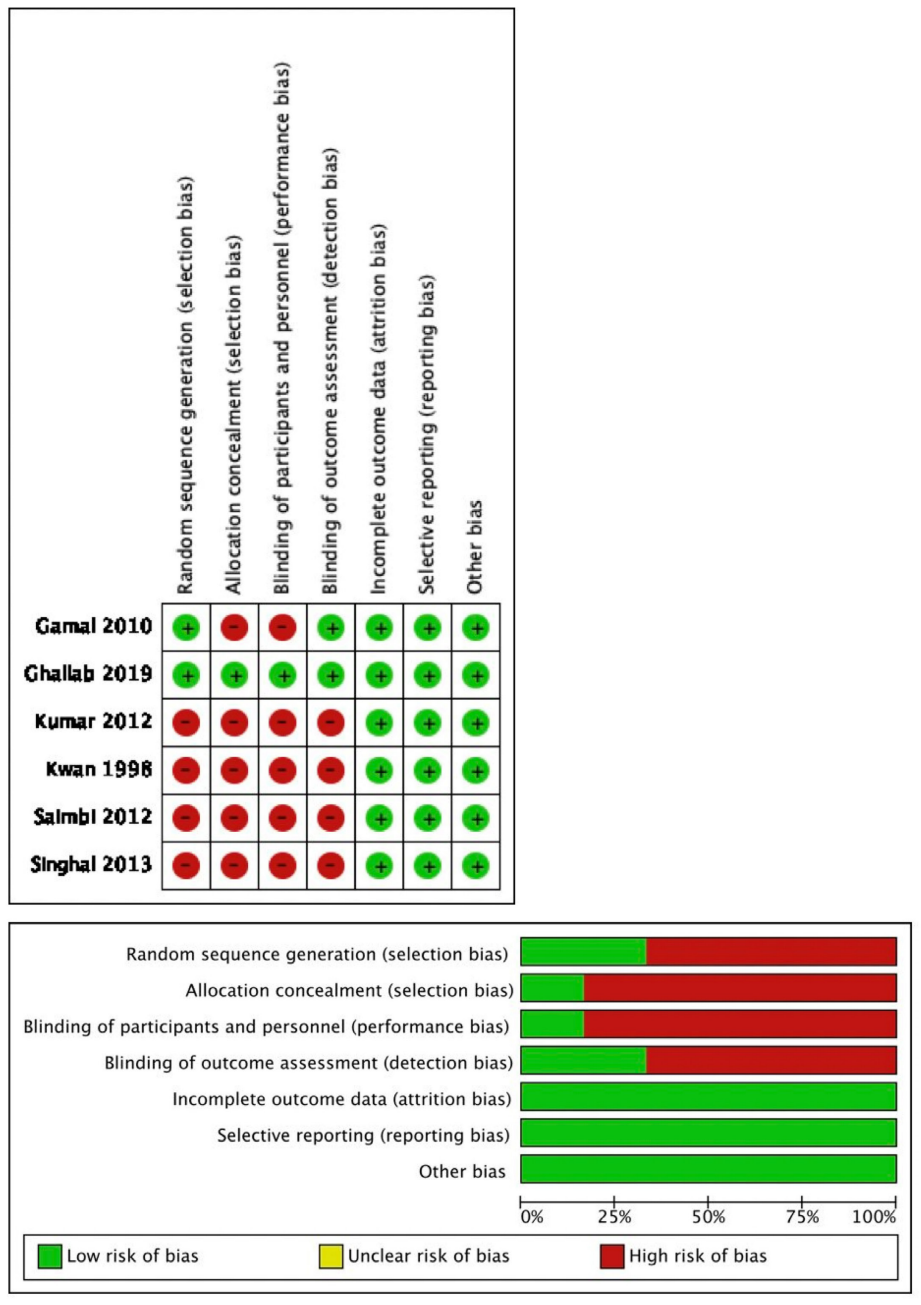
Risk of bias of the included studies.

**Figure 2A:**
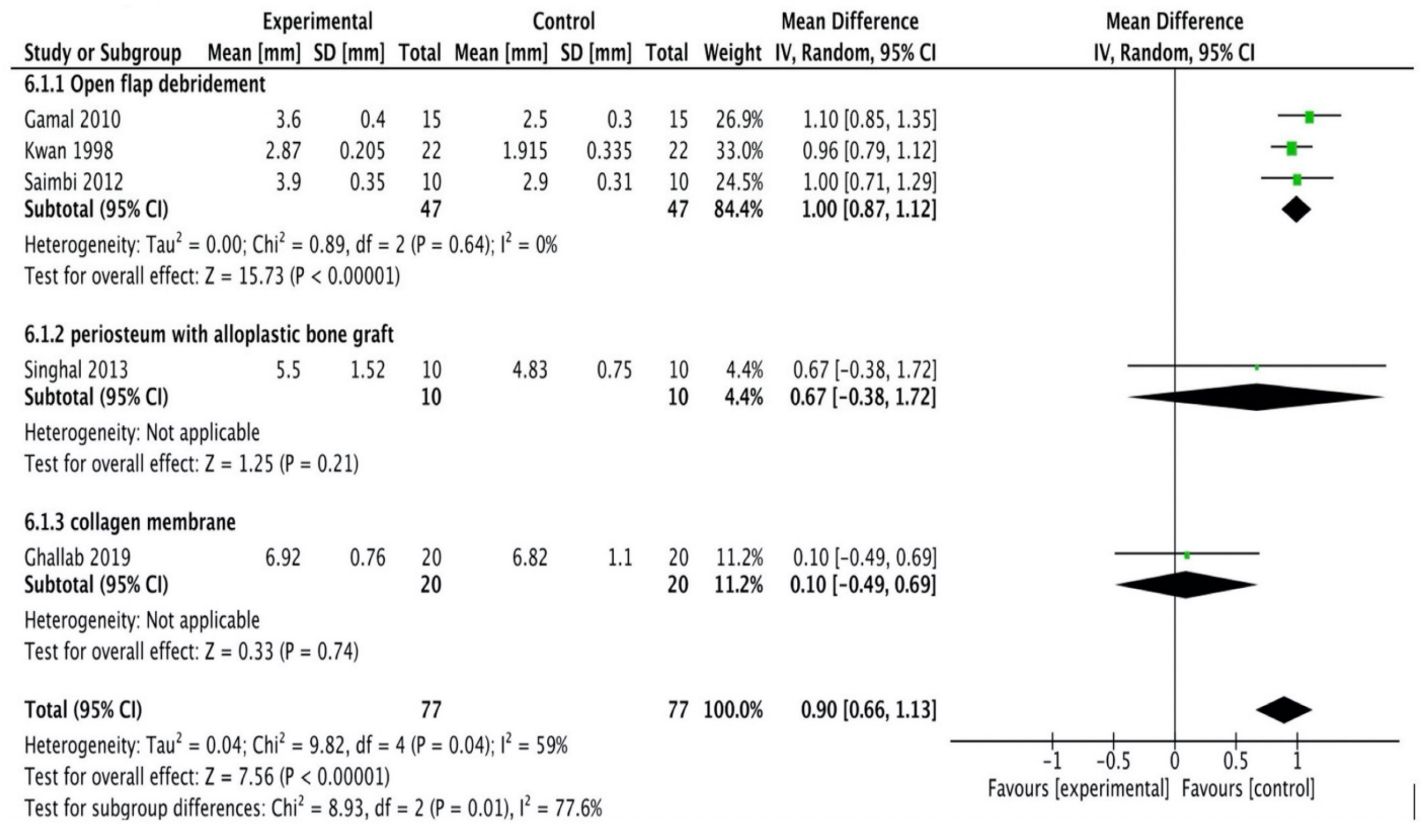
Forest plot for probing depth.

**Figure 2B:**
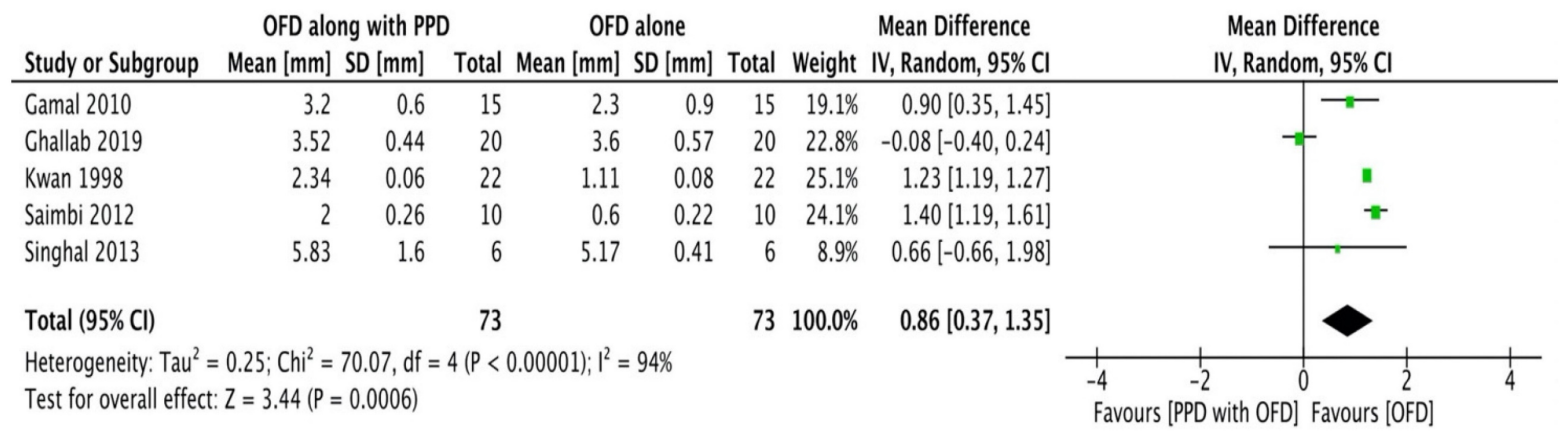
Forest plot for clinical attachment levels.

**Figure 2C:**
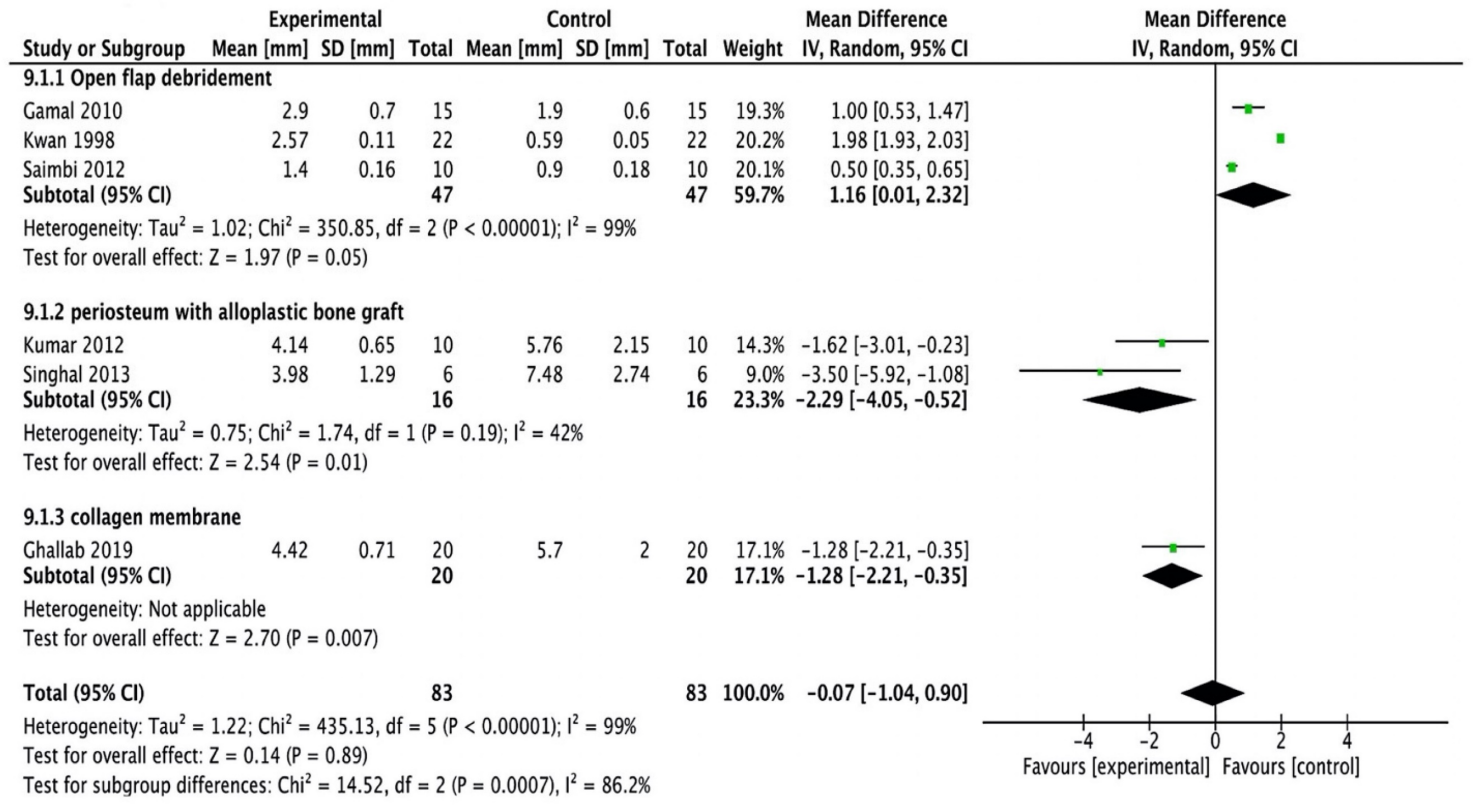
Forest plot for bone defect fill.

**Figure 2D:**
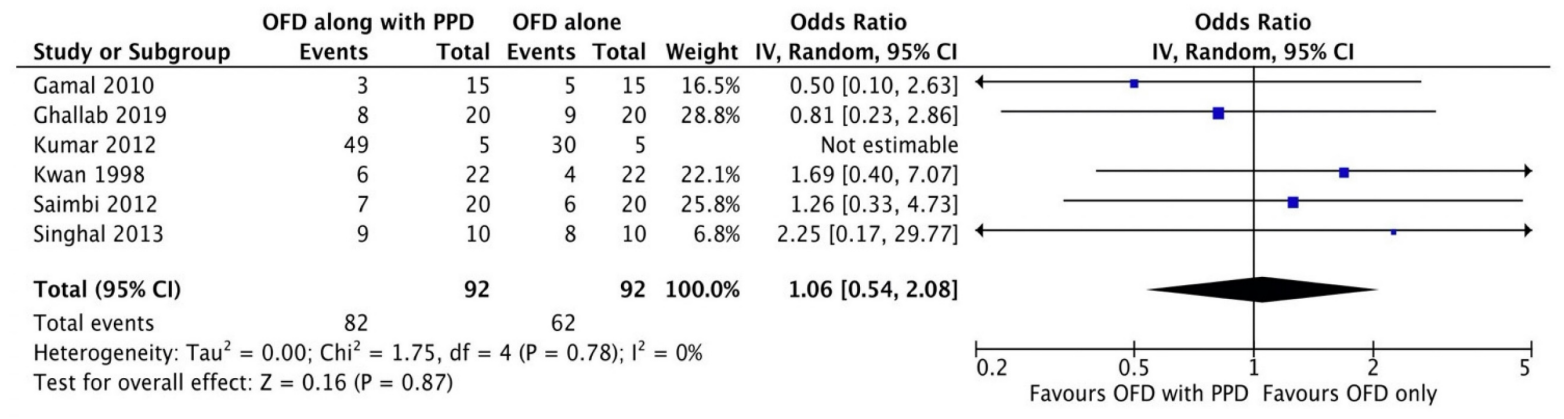
Forest plot for gingival recession.

**Figure 2E:**
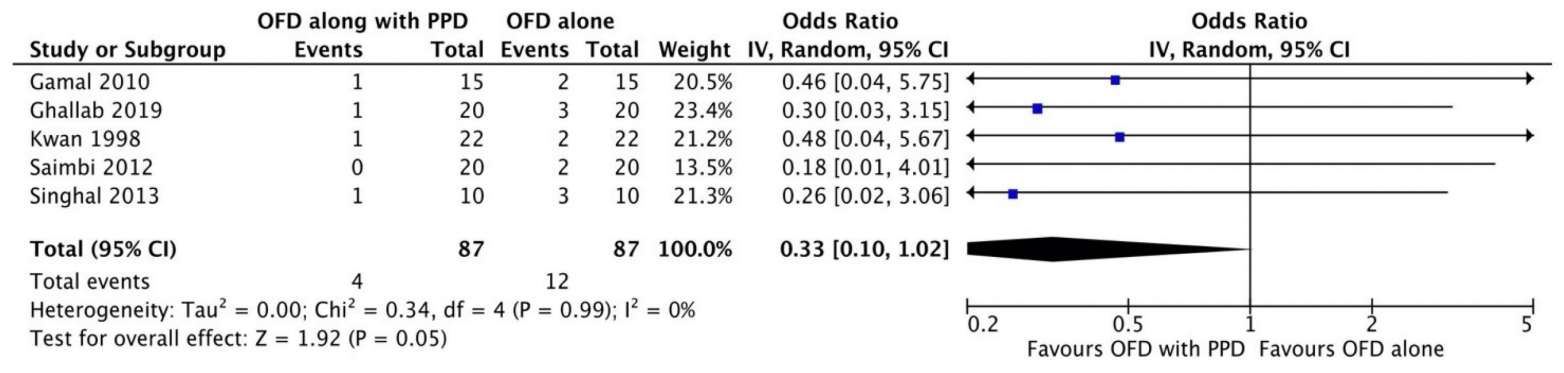
Forest plot for plaque index.

**Figure 2F:**
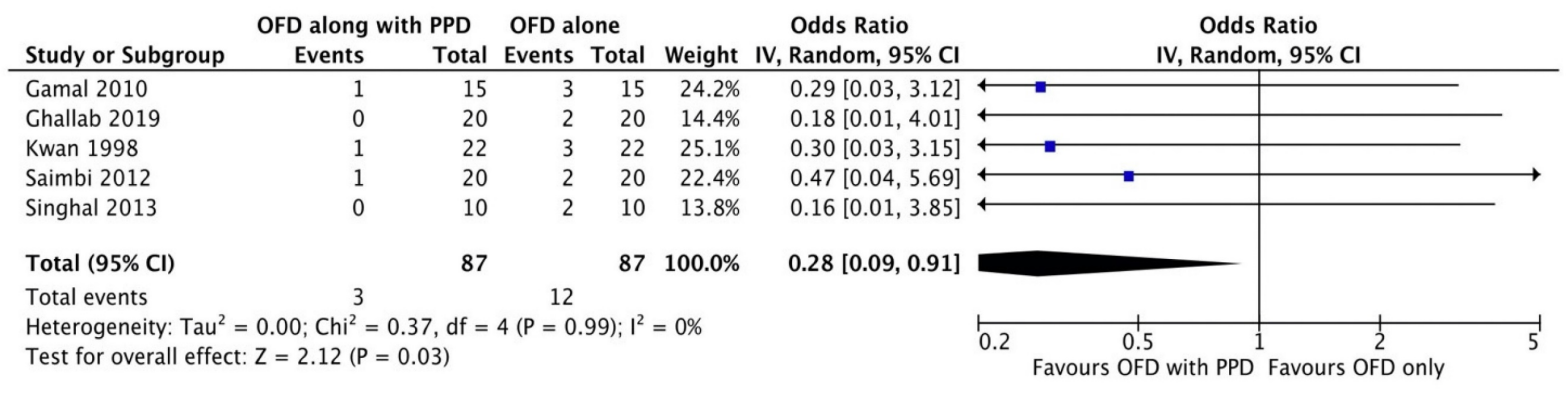
Forest plot for gingival index.

**Figure 2G:**
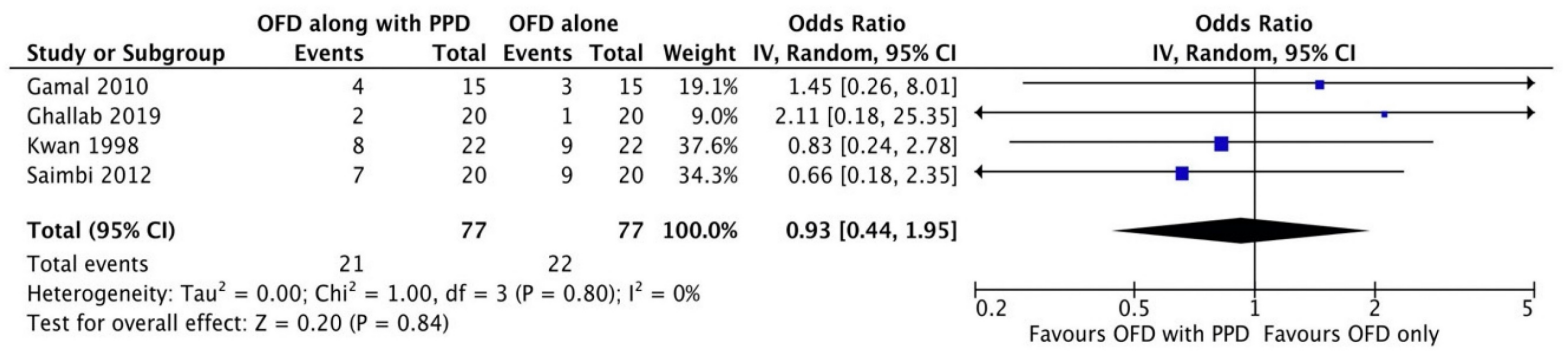
Forest plot for bleeding on probing.

**Table 1:** General information of the included studies

Author (year and place)	Journal	Age range	Total participants	Follow up	Intervention	
					Control	Test
Kwan et al. 1998, LA California	Journal of Periodontology	48.78 years ± 10.54	22 patients	6 months	OFD+ Periosteal graft as a barrier membrane	Open flap debridement
Kumar et al. 2012, India	Journal of Indian Society of Periodontology	18 to 50 years	10 patients	9 months	periosteum as a barrier membrane	Open flap debridement with periosteum and an alloplastic graft
Singhal et al. 2013, India.	Journal of Periodontology	20 to 50 years old	20 patients (12 males and 8 females)	6 months	Periosteum as a barrier membrane	Open flap debridement with periosteum and an alloplastic graft
Saimbi et al. 2013, India	Journal of Indian Society of Periodontology	20-50 years	10 patients (20 sites)	3 months	Periosteum as a barrier membrane	Open Flap debridement
Gamal et al. 2010, Cairo Egypt	Journal of the International Academy of Periodontology	27 to 45 years	15 patients	3, 6, 9 months	Periosteal pedicle graft as a barrier membrane	Open Flap debridement
Ghallab et al. 2019, Egypt	Egyptian Dental Journal	35 to 50 years	20 patients	6 months	Periosteal pedicle graft as a barrier membrane	Open flap debridement with collagen membrane

**Table 2:** Data extracted from the included studies

Reference	MD in PD between baseline and follow-up	MD in CAL between baseline and follow-up	MD in BDF between baseline and follow-up	MD in PI between baseline and follow-up	MD in GI between baseline and follow-up	MD in GR between baseline and follow-up	MD in BOP between baseline and follow-up
Kwan et al., 1998,	4.12 0.66	2.42 ± 0.34	2.70 ± 0.48	0.56 ± 0.22	-	0.42 ± 0.33	0.50 0.22
Kumar et al., 2012	5.76±2.15	-	-	-	-	-	-
Singhal et al., 2013	5.50	5.83	7.48	-	-	-	-
Saimbi et al., 2013	3.90±0.35	2.00 ± 0.26	1.40 ± 0.16	-	-	-	-
Gamal Y. et al., 2010	6.4 ± 1.4	4.9 ± 1.1	4.3 ± 0.7	0.9 ± 0.5	1.7 ± 0.5	2.9± 0.7	66.7
Ghallab et al., 2019	3.17±0.65	3.52±0.44	3.94±4.09	0.50 ±0.53	0.33 ± 0.52	-	-

MD= mean difference; PD= probing depth, CAL= clinical attachment level, BDF= bone defect fill, PI= plaque index, GI= gingival index, GR= gingival recession and BOP= bleeding on probing
